# Combining serum calcitonin, carcinoembryonic antigen, and neuron‐specific enolase to predict lateral lymph node metastasis in medullary thyroid carcinoma

**DOI:** 10.1002/jcla.23278

**Published:** 2020-03-06

**Authors:** Liuqing Ye, Xi Zhou, Jie Lu, Yanzhong Wang, Xinyou Xie, Jun Zhang

**Affiliations:** ^1^ Clinical Laboratory Sir Run Run Shaw Hospital School of Medicine Zhejiang University Hangzhou China; ^2^ Institute of Cancer and Basic Medical (ICBM) Chinese Academy of Sciences Hangzhou China; ^3^ Department of Clinical Laboratory Cancer hospital of the University of Chinese Academy of Sciences Hangzhou China; ^4^ Department of Clinical Laboratory Zhejiang cancer hospital Hangzhou China; ^5^ Biomedical Research Center Sir Run Run Shaw Hospital School of Medicine Zhejiang University Hangzhou China

**Keywords:** calcitonin, carcinoembryonic antigen, lymph node metastasis, medullary thyroid carcinoma, neuron‐specific enolase

## Abstract

**Background:**

This study aimed to investigate the clinical application of combined detection of serum calcitonin (Ctn), carcinoembryonic antigen (CEA), and neuron‐specific enolase (NSE) in predicting lateral lymph node metastasis (LLNM) in medullary thyroid carcinoma (MTC).

**Methods:**

Seventy‐four consecutive patients with MTC were enrolled. The relationship between serum Ctn, CEA, and NSE and LLNM was retrospectively analyzed by univariate analysis and logistic regression analysis. Furthermore, the clinical application of serum Ctn, CEA, and NSE combined detection in prediction of LLNM in MTC was also evaluated.

**Results:**

The rate of LLNM in this study was 48.64% (36/74).The expression levels of serum Ctn, CEA, and NSE in MTC with LLNM were significantly higher than those without LLNM (all *P* < .01). The area under the curve (AUC) predicted by serum Ctn, CEA, and NSE for LLNM in MTC patients was 0.867, 0.831, and 0.726, respectively, and the AUC of serum Ctn, CEA, and NSE combined detection was up to 0.890, higher than using a single biomarker. The sensitivity and specificity of serum Ctn, CEA, and NSE combined detection in prediction of LLNM were 88.89% and 81.57%, respectively.

**Conclusions:**

The concentrations of serum Ctn, CEA, and NSE are closely related to LLNM in MTC, and the combined detection of all three biomarkers has a higher clinical value in the evaluation of MTC patients with LLNM. With more perspective study in the future, it would be an indicator of influencing personalized surgical strategy for different MTC patients.

## 1 INTRODUCTION

Medullary thyroid carcinoma (MTC) is a neuroendocrine neoplasm originating from thyroid parafollicular C cells and accounts for only 5% of all thyroid cancers, but its malignancy is relatively high, causing 8%‐13% of thyroid cancer‐related deaths due to its aggressiveness.^1^ It has been reported that cervical lymph node metastasis is a common occurrence in MTC and more than 50% of patients with MTC have cervical lymph node metastasis at the time of initial diagnosis. Moreover, cervical lymph node metastasis plays an important role in evaluating the therapeutic effect and prognosis for MTC.^2^ Therefore, the status of cervical lymph nodes in MTC patients should be carefully evaluated before treatment.Surgery is the only curative treatment for MTC. The treatment of cervical lymph nodes in current clinical practice includes the dissection of central and lateral cervical lymph nodes. Nowadays, central lymph node dissection is recommended for MTC patients according to the American Thyroid Association (ATA) guideline; however, it is still controversial whether lateral neck dissection is necessary for all the MTC patients.^3^, ^4^

Previous studies reported that MTC can synthesize and secrete a variety of bioactive substances, such as calcitonin (Ctn), carcinoembryonic antigen (CEA), and neuron‐specific enolase (NSE).^5^, ^6^, ^7^, ^8^, ^9^ Although numerous reports have shown that the expression levels of Ctn and CEA are related to cervical lymph node metastasis, the relationship between combined detection of serum markers and lateral lymph node metastasis (LLNM) in MTC patients is rarely studied. In this study, we aim to analyze the differences of serum markers between MTC patients with LLNM (Group LLNM) and MTC patients without LLNM (Group non‐LLNM), and to investigate the clinical application of combined detection of serum markers in predicting LLNM of MTC.

## 2 MATERIALS AND METHODS

### 2.1 Patients

We reviewed the medical records of 74 patients with MTC at Zhejiang Cancer Hospital from January 2013 to April 2019. Inclusion criteria were as follows: (a) No neck surgery or other treatments were received before admission; (b) primary lesion resection and neck dissection were performed in all patients; (c) postoperative and histopathological MTC; and (d) the clinical data were complete. Exclusion criteria were as follows: (a) No neck dissection was performed; (b) patients with infection, hypohepatia, renal insufficiency, and hyperparathyroidism; (c) patient with malignant tumors of other organs; and (d) preoperative serum markers were not detected or incomplete. This study was approved by the Ethics Committee of Zhejiang Cancer Hospital, and all patients or their guardians provided the written informed consent.

### 2.2 Operation principle

Cervical lymph node dissection was performed during thyroidectomy. Principle of thyroidectomy: Patients with hereditary MTC, tumor size > 1 cm, extraglandular invasion, bilateral or multi‐focus, and bilateral cervical lymph node metastasis were performed with total thyroidectomy, while patients with unilateral intrathyroidal tumors and tumor size ≤ 1 cm were treated with hemithyroidectomy and isthmectomy. Principle of cervical lymph node dissection: Central lymph nodes were routinely dissected, and lateral compartment (levels II‐V) with evidence of lymph node metastasis should be dissection. The upper mediastinal lymph nodes (level VII) should be also cleaned if lymphadenopathy was present according to neck imaging. The recurrent laryngeal nerves should be directly visualized throughout the nodal dissection in order to avoid injury. The parathyroid gland and its blood supply should be retained.

### 2.3 Detection of serum markers

Fasting blood samples were drawn from all patients before surgery in the morning and transported to our laboratory within 3 hours after phlebotomizing. After centrifuging at 3000 r/min for 10 minutes, serum was separated and analyzed. All specimens should be without jaundice, hemolysis and lipid. Serum Ctn was measured via Siemens IMMULITE^®^ 2000 automatic chemiluminescence immunoassay analyzer using a standard assay kit for in vitro diagnosis (Siemens Healthcare Diagnostics Products Limited); serum alpha‐fetoprotein (AFP), carbohydrate‐associated antigen 19‐9 (CA19‐9), and CEA were measured by Siemens Centaur^®^ XP automatic chemiluminescence immunoassay analyzer and its supporting reagents (Siemens Healthcare Diagnostics Inc); serum cytokeratin 19 fragment (CYFRA21‐1) and NSE were measured by Roche Cobas e 602 automatic electrochemical luminescence immunoanalyzer and its matched reagents (Roche Diagnostics GmbH); and serum carbohydrate‐associated antigen 242 (CA242) levels were measured by Shenzhen New Industries MAGLUMI 2000 automatic chemiluminescence immunoassay analyzer and its supporting reagents (Shenzhen New Industries Biomedical Engineering Co., Ltd.). All testing procedures were performed in accordance with the instructions provided by each manufacturer.

### 2.4 Pathological examination

The surgical specimens were diagnosed by two pathologists. The histological type, lesion numbers, tumor size, and capsular invasion were carefully evaluated. The lymph node specimens were divided strictly, including central, lateral, and upper mediastinal lymph nodes. Immunohistochemistry was used to make the diagnosis of MTC if the case was difficult to determine by routine pathology techniques.

### 2.5 Statistical analysis

All the statistical analyses were performed using the SPSS package version 22.0 (SPSS Inc). Data were displayed as mean and standard deviation (MSD), median and quartile (*P50*, [*P25*, *P75*]), and count (percentage). Quantitative variables were compared between two groups of independent samples using Wilcoxon test, and the correlation analysis was performed by Spearman test. Logistic regression analysis was conducted for establishing a model of serum markers to predict LLNM in MTC. The discriminative power of the predictive value was assessed by area under the receiver operating characteristic (ROC) curves. *P*‐value < .05 was considered statistically significant.

## 3 RESULTS

### 3.1 Clinical characteristics of MTC patients

From January 2013 to April 2019, the 74 consecutive patients (39 males and 35 females, mean age 51.38 ± 12.89 years) underwent surgery for untreated MTC in our center were enrolled. The patients’ baseline characteristics are listed in Table 1. Cervical lymph node metastasis in our cohort occurred in 44 (59.46%) patients (N1), 43 (58.11%) of whom had central lymph node metastasis (N1a), while 36 (48.64%) of whom had lateral lymph node metastasis (N1b).

**Table 1:** Clinical characteristics of patients

Patient characteristics	n/mean ± SD
Number of patients	74
Age at diagnosis, mean ± SD, y	51.38 ± 12.89
Gender, male/female	39/35
Largest tumor size, mean ± SD, cm	2.07 ± 1.56
Bilateral carcinoma, n (%)	11 (14.86%)
Extrathyroidal infiltration, n (%)	31 (41.89%)
Cervical lymph node metastases (N1), n (%)	44 (59.46%)
Central lymph node metastases (N1a), n (%)	43 (58.11%)
Lateral lymph node metastases (N1b), n (%)	36 (48.64%)

Abbreviation: M ± SD, mean ± standard deviation.

### 3.2 Comparison of serum markers in two groups of MTC patients

Patients with LLNM were classified as the Group LLNM, and the other patients were classified as the Group non‐LLNM. Table 2 shows the relationship between expression levels of serum markers and LLNM in MTC patients. The expression levels of serum Ctn, CEA, and NSE in Group LLNM were significantly higher than the Group non‐LLNM (all *P* < .01). However, there was no significant difference in the levels of serum AFP, CYFRA21‐1, CA19‐9, and CA242 between the two groups (all *P* > .05). Spearman analysis showed that the levels of serum Ctn, CEA, and NSE in patients with MTC were positively correlated with each other (*r_Ctn‐CEA_* = 0.871, *r_Ctn‐NSE_* = 0.407, *r_CEA‐NSE_* = 0.465, all *P* < .001).

**Table 2:** Relationship between LLNM and expression levels of serum markers in MTC patients (*P50*, [*P25*, *P75*])

Variable	Group LLNM (n = 36)	Group non‐LLNM (n = 38)	*Z*	*P*
Ctn (pg/mL)	2000.00 (906.00, 2000.00)*	130.80 (33.85, 393.08)	−5.566	<.001
CEA (ng/mL)	92.53 (24.16, 236.07)	8.33 (2.89, 26.91)	−4.894	<.001
NSE (ng/mL)	14.45 (11.23, 16.95)	11.80 (10.48, 12.56)	−3.337	.001
AFP (ng/mL)	2.77 (2.28, 3.92)	2.69 (2.05, 4.57)	−0.265	.791
CYFRA21‐1 (ng/mL)	2.60 (2.08, 4.04)	1.91 (1.39, 3.38)	−1.741	.082
CA19‐9 (U/mL)	6.54 (4.43, 16.77)	7.69 (3.77, 14.79)	−0.438	.661
CA242 (U/mL)	3.97 (2.60, 7.30)	4.86 (2.61, 6.71)	−0.227	.820

Abbreviations: AFP, alpha‐fetoprotein; CA19‐9, carbohydrate antigen; CA242, carbohydrate antigen 242; CEA, carcinoembryonic antigen; Ctn, calcitonin; CYFRA21‐1, cytokeratin 19 fragment; LLNM, lateral lymph node metastasis; MTC, medullary thyroid carcinoma; NSE, neuron‐specific enolase.

* The upper limit of serum CT detection is 2000.

### 3.3 Clinical value of serum Ctn, CEA, and NSE combined detection to predict LLNM in MTC patients

**Figure 1:**
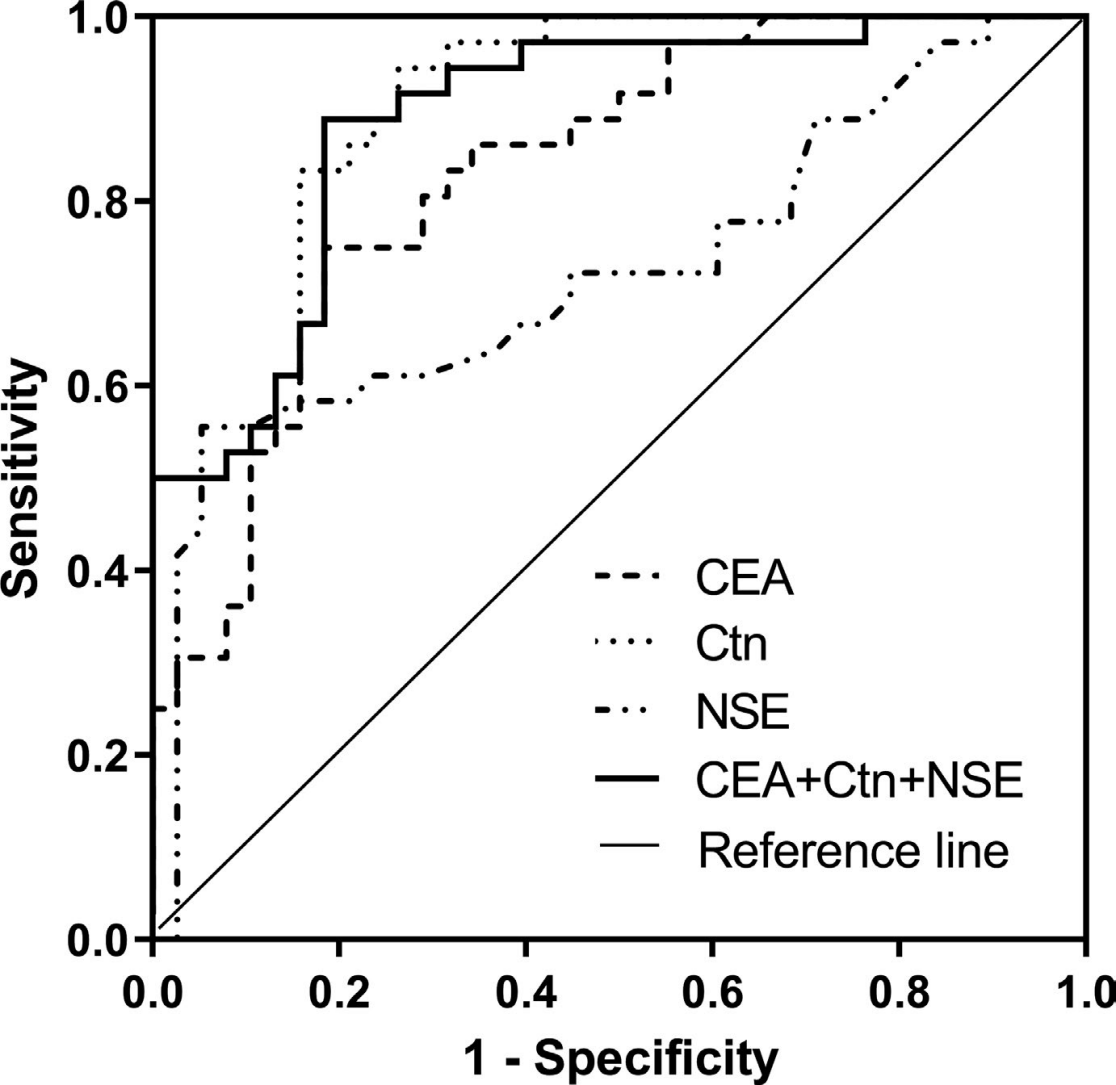
Receiver operating characteristic curves of serum Ctn, CEA, and NSE combined detection for LLNM in MTC patients

Univariate analysis above showed that serum Ctn, CEA, and NSE were related with LLNM in MTC patients. Thus, we derived the following prediction equation of serum markers combined detection for LLNM in MTC patients by logistic regression analysis (enter method) (as shown in Table 3): Logistic (P) = −3.5390.002 × Ctn + 0.001 × CEA + 0.151 × NSE. To evaluate the predictive value of serum markers for LLNM, ROC curves were performed (as shown in Table 4 and Figure 1). The AUC of serum Ctn for predicting LLNM in MTC patients was 0.867 (95% CI: 0.778‐0.956), which was higher than that of CEA and NSE, respectively. (AUC*_CEA_* = 0.831, 95% CI: 0.739‐0.922; AUC*_NSE_* = 0.726, 95% CI: 0.606‐0.845). The AUC of the three markers combined detection was 0.890 (95% CI: 0.816‐0.963), which was the highest. When the Youden index had a maximum value, the sensitivity and specificity of combined detection of serum Ctn, CEA, and NSE for predicting LLNM were 88.89% and 81.57%, respectively.

**Table 3:** Logistic regression analysis of serum Ctn, CEA, and NSE combined detection to predict LLNM in MTC patients

Variable	B	SE	Wald	*P*	OR (95% CI)
Ctn	0.002	0.000	12.256	.000	1.002 (1.001‐1.002)
CEA	0.001	0.002	0.195	.658	1.001 (0.997‐1.005)
NSE	0.151	0.098	2.373	.1123	1.163 (0.960‐1.410)
Constant	−3.539	1.299	7.419	.006	0.029

Abbreviations: CEA, carcinoembryonic antigen; Ctn, calcitonin; LLNM, lateral lymph node metastasis; MTC, medullary thyroid carcinoma; NSE, neuron‐specific enolase.

**Table 4:** Prediction value of serum Ctn, CEA, and NSE combined detection for LLNM in MTC patients

Variable	ROC‐AUC	SE	*P*	Cutoff value	Sensitivity (%)	Specificity (%)
Ctn	0.867 (0.778‐0.956)	0.045	.000	303.10 (pg/mL)	94.44	73.68
CEA	0.831 (0.739‐0.922)	0.047	.000	29.68 (ng/mL)	75.00	81.57
NSE	0.726 (0.606‐0.845)	0.061	.001	13.95 (ng/mL)	55.56	94.74
CEA + Ctn + NSE	0.890 (0.816‐0.963)	0.037	.000	0.30	88.89	81.57

Abbreviations: CEA, carcinoembryonic antigen; Ctn, calcitonin; LLNM, lateral lymph node metastasis; MTC, medullary thyroid carcinoma; NSE, neuron‐specific enolase.

## 4 DISCUSSION

Cervical lymph node metastasis is an important factor of prognosis in MTC patients. Once the patients have lymph node metastasis in lateral compartment (level II to V), it should be classified as phase IV, which causes poor prognosis, and lateral neck lymph node dissection is then required. It has been reported that the rate of LLNM in MTC patients was 40%‐66.7%.^10^, ^11^ This study found that the metastasis rate of lateral compartment was 48.64%, which was consistent with the previous literatures. Therefore, Iit is necessary to carefully evaluate the status of lateral lymph node in MTC patients before surgical operation. Ultrasound examination and computed tomography of the neck are recommended for patients with the extensive neck disease of MTC,^1^ but their sensitivity is not ideal.^12^ The purpose of this study was to investigate the clinical application value of serum markers in prediction of LLNM in MTC. Our data showed that the expression levels of serum Ctn, CEA, and NSE in MTC patients with LLNM (Group LLNM) were significantly higher than those without LLNM (Group non‐LLNM), whereas the levels of serum AFP, CYFRA21‐1, CA19‐9, and CA242 had no statistical difference between these two groups. Spearman correlation analysis showed that the levels of serum Ctn, CEA, and NSE in MTC were positively correlated with each other, indicating that the levels of serum Ctn, CEA, and NSE might be helpful for the evaluation of LLNM in MTC patients.

Ctn is a type of polypeptide hormone consisting of 32 amino acids which is released by thyroid parafollicular C cells with a molecular weight of 3418 Dalton. It was firstly demonstrated by Copp et al^13^ in 1962 and proposed that Ctn can reduce blood calcium level. Subsequent studies found that serum Ctn was a specific marker of MTC, and preoperative serum Ctn levels could reflect the tumor burden of MTC, including tumor size, lymph node metastasis, number of metastasis, and TNM stage, etc.^14^, ^15^ Machens et al^16^ showed preoperative serum Ctn levels could be used to predict the different locations of lymph node metastasis. Lymph node metastases were present in the ipsilateral central and lateral neck, contralateral central neck, contralateral lateral neck, and upper mediastinum, respectively, beyond basal Ctn thresholds of 20, 50, 200, and 500 pg/mL. Our study indicates serum Ctn level in patients with MTC is closely related to LLNM. When serum Ctn levels were beyond threshold levels of 303.10 pg/mL, the AUC of predicting LLNM was up to 0.867, with sensitivity and specificity of 94.44% and 73.68%, respectively. Therefore, serum Ctn should be recommended clinically to facilitate the evaluation of LLNM for MTC. Serum CEA, as a broad‐spectrum tumor marker, may increase in a variety of tumors, including gastrointestinal malignancies, lung cancer, and breast cancer.^17^, ^18^ Previous researches had found that MTC can also secrete CEA, and the increased level of serum CEA in MTC patients was synchronous with Ctn.^9^, ^19^ This study also showed that there was significant correlation between serum CEA and Ctn in MTC patients (rct‐cea = 0.871). It was reported that the positive expression rate of CEA in MTC was also very high, and its concentration was closely related to the progression and prognosis of MTC, which has increasingly become an important indicator in the diagnosis and treatment of MTC.^5^, ^8^, ^9^ A recent Canadian study of Canadian also reported that preoperative CEA level was closely related to tumor size, TNM stage, regional lymph node metastasis, biochemical cure, and survival rate.^9^ The results of our study demonstrate that serum CEA in MTC patients with LLNM was significantly higher than that in the group without LLNM. When serum CEA levels were beyond threshold levels of 29.68 ng/mL, the AUC of predicting LLNM was up to 0.831, with sensitivity and specificity of 75.00% and 81.57%, respectively. Since CEA is commonly used in clinical practice, we recommend serum CEA as an important indicator to evaluate LLNM of MTC.

NSE is an important tumor marker in the serum of patients with lung cancer.^20^, ^21^ Elevated serum NSE level is also associated with neuroblastoma, neuroendocrine neoplasms, renal cell carcinoma, and other diseases.^22^, ^23^ As a neuroendocrine tumor, we hypothesized that MTC could also secrete NSE theoretically. However, there are few reports about the study of serum NSE in MTC. In this study, our data revealed that the median level of NSE was 14.45 (11.23, 16.95) ng/mL in Group LLNM and 11.80 (10.48, 12.56) ng/mL in Group non‐LLNM, respectively. Differences between the two groups had statistical significance (*P* = .001), indicating that the level of NSE is also closely related to LLNM of MTC. Furthermore, we find that when 13.95 ng/mL was used as the threshold, the AUC for predicting LLNM was 0.726, with a relatively low sensitivity (55.6%) but a high specificity of 94.7%. Thus, we believe that measurement of serum NSE can be used as an auxiliary marker for predicting LLNM in MTC.

Based on the advantages of the above‐mentioned serum markers, we attempted to establish a prediction model for LLNM of MTC using the logistic regression analysis. The results showed that the AUC of serum Ctn, CEA, and NSE combined to predict the LLNM of MTC was the highest (0.890), with a sensitivity of 88.89% and a specificity of 81.57%. Therefore, we recommend that these three markers can be combined to improve the accuracy of evaluating LLNM in MTC.

In conclusion, the expression levels of serum Ctn, CEA, and NSE are closely related to LLNM in MTC, and the combined detection of the three markers has a higher clinical significance in the evaluation of MTC patients with LLNM. It provided some indications for the extent of cervical lymph node dissection in the operation of MTC.

However, there are still some limitations in this study. Due to the retrospective analysis, there may be an inevitable selection bias. Prognosis analysis and a larger cohort study will be needed in the future to verify our findings.
